# Supplementary material for: Hedgehog and Bmp signaling pathways play opposing roles during establishment of the cardiac inflow tract in zebrafish
Source: Development. 2026 Jan 22;153(2):dev205111. doi: 10.1242/dev.205111 (PMC12863301; doi:10.1242/dev.205111)
Supplement: Supplementary information [file develop-153-205111-s1.pdf]

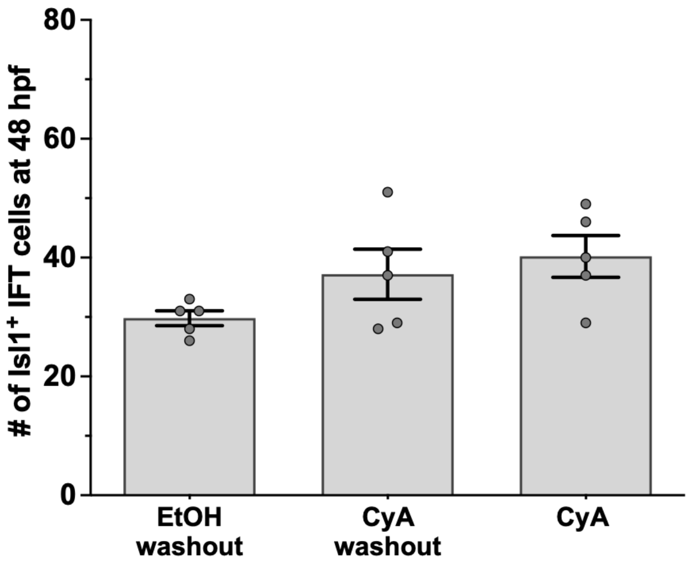

Fig. S1. Embryos treated with CyA during gastrulation display a trend toward an increased number of IFT cardiomyocytes

Graph indicates the number of Isl1+ cardiomyocytes in the IFT at 48 hpf. Embryos were treated with ethanol or CyA beginning at sphere stage and ending at 1 s when the compound was washed out ("EtOH washout" and "CyA washout") or with CyA beginning at sphere stage and ending at 48 hpf ("CyA"). Embryos treated with CyA during either of these intervals exhibited a trend toward an increased number of Isl1+ IFT cardiomyocytes compared to the number found in ethanol-treated controls.

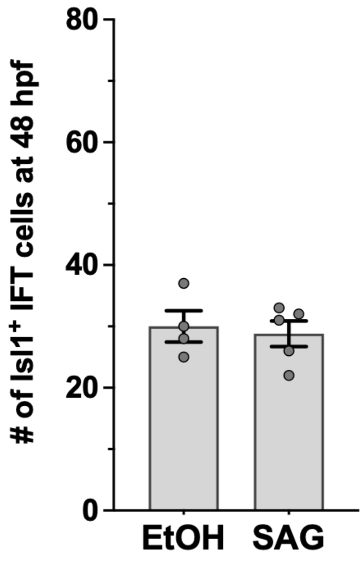

Fig. S2. IFT cardiomyocyte number is unaffected by treatment with the Smo agonist SAG

Graph indicates the number of Isl1+ cardiomyocytes in the IFT at 48 hpf. Embryos treated with SAG beginning at dome stage show no significant difference in the number of Isl1+ IFT cardiomyocytes, compared to ethanol-treated controls. We note that this concentration of SAG has previously been demonstrated to be effective at activating the Hh signaling pathway (Muthu et al., 2016; Burton et al., 2022).

A

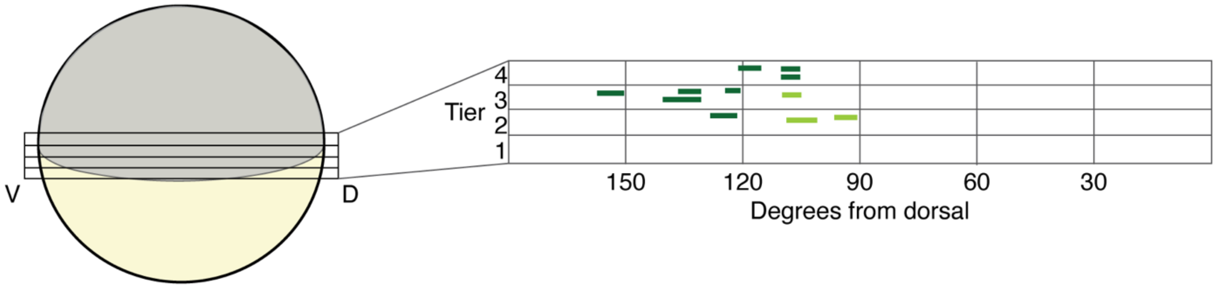

B

Tier	Angle	Side	Progeny
2	92-98	L	AC and IFT
2	101-109	L	AC and IFT
2	122-129	L	IFT
3	105-110	L	AC and IFT
3	121-125	L	IFT
3	130-136	R	IFT
3	130-140	R	IFT
3	153-160	L	IFT
4	105-110	L	IFT
4	105-110	L	IFT
4	115-121	R	IFT

Fig. S3. IFT progenitors originate from a ventral portion of the lateral margin

(A) In our previous experiments (Keegan et al., 2004; Keegan et al., 2005), fate mapping of cardiac progenitors was performed by injecting a caged fluorescein-dextran lineage tracer at the single-cell stage, followed by uncaging to label small groups of blastomeres at 40% epiboly before analysis of cell fates at 48 hpf. For each set of labeled cells, their location at 40% epiboly was recorded in terms of latitude, using cell tiers to measure distance from the margin, and longitude, using degrees from the dorsal midline. Our retrospective analysis of these data evaluated whether any labeled cardiomyocytes were found in the bottom 30% of the atrium at 48 hpf (see Materials

Development • Supplementary information

and Methods). As this region approximates the location of Isl1+ IFT cardiomyocytes, we classified these examples as instances of IFT progeny, whereas labeled cells elsewhere in the atrium were classified as instances of atrial cardiomyocyte (AC) progeny. Altogether, we identified eight examples (marked in dark green) in which labeled cells appeared to give rise to IFT progeny. We also found an additional three examples (light green) in which labeled cells appeared to give rise to both IFT and AC progeny. In all 11 examples, IFT progenitors were located in tiers 2-4, between 92 and 160 degrees from dorsal.

(B) Table lists each instance of IFT labeling, indicating the cell tier, degrees from dorsal, and side (left or right indicated by L or R, respectively) at 40% epiboly, as well as the final myocardial contribution of the labeled progeny at 48 hpf. We do not observe any asymmetry between the left and right sides of the embryo in this set of data: of all experiments that resulted in labeled atrial cells, we observe labeled IFT cardiomyocytes 73% (8/11) of the time on the left side of the embryo and 75% (3/4) of the time on the right side of the embryo.

Development • Supplementary information

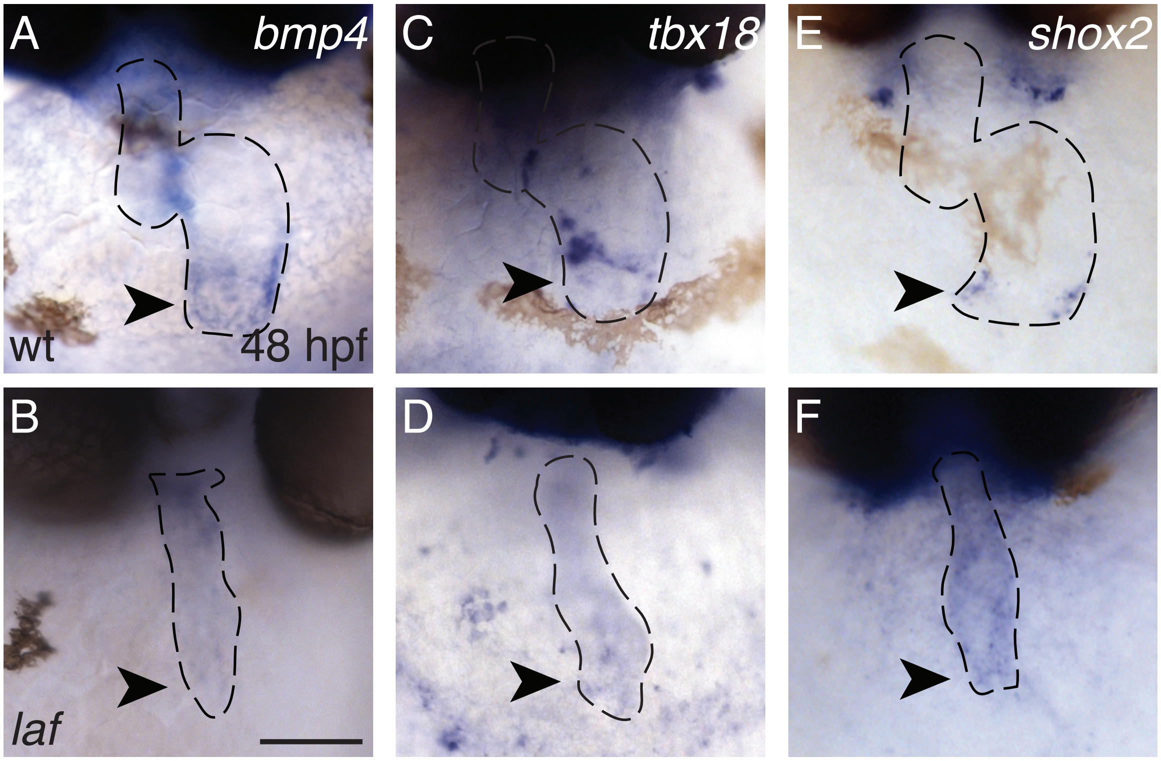

Fig. S4. Bmp signaling promotes expression of IFT markers (A-F) In situ hybridization depicts expression of *bmp4* (A,B), *tbx18* (C,D) and *shox2* (E,F) at 48 hpf. Frontal views; arrowheads indicate the IFT. Whereas wt embryos (A,C,E) show discrete expression of IFT markers in the venous pole, concentrated expression of *bmp4* (B), *tbx18* (D), and *shox2* (F) is not evident at the venous pole of most *laf* mutant embryos (n=44 wt, 29/52 *laf* for *bmp4;* n=11 wt, 10/11 *laf* for *tbx18;* n=13 wt, 14/14 *laf* for *shox2*). Note that variability observed in the *laf* mutant phenotype may reflect a variable degree of maternal *acvr1l* contribution in individual embryos (Mintzer et al., 2001). Scale bar: 50 µm.

Development • Supplementary information

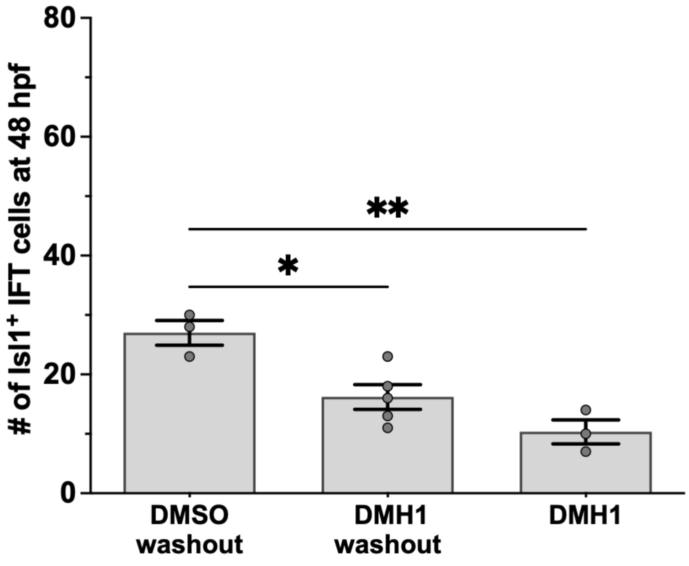

Fig. S5. IFT cardiomyocyte number is reduced in embryos treated with DMH1 during gastrulation

Graph indicates the number of Isl1+ cardiomyocytes in the IFT at 48 hpf. Embryos were treated with DMSO or DMH1 beginning at sphere stage and ending at 1 s when the compound was washed out ("DMSO washout" and "DMH1 washout") or with DMH1 beginning at sphere stage and ending at 48 hpf ("DMH1"). Embryos treated with DMH1 during either of these intervals had significantly fewer Isl1+ IFT cardiomyocytes than were found in DMSO-treated controls. *p<0.05; **p<0.01.

Development • Supplementary information

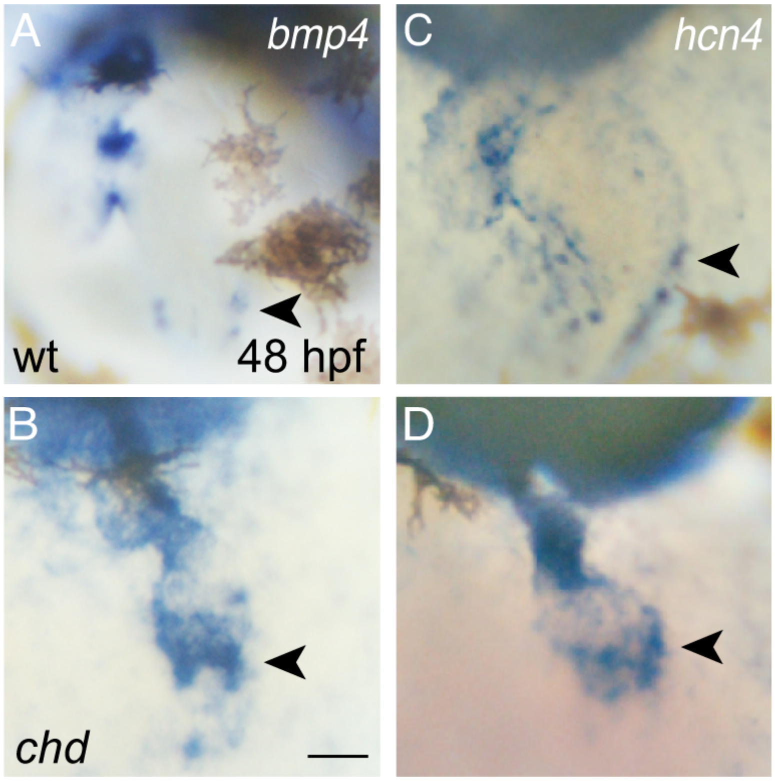

Fig. S6. Expanded expression of IFT markers in *chd* mutants (A-D) In situ hybridization depicts expression of *bmp4* (A,B) and *hcn4* (C,D) at 48 hpf in wt (A,C) and *chd* mutant embryos (B,D). Frontal views; arrowheads indicate the IFT. Broader expression of *bmp4* is visible at the IFT in *chd* mutants (n=21), compared to the expression at the IFT in wt siblings (n=24). Expression of *hcn4* expression is similarly expanded in *chd* mutants (n=16), compared to wt siblings (n=19). Scale bar: 30 µm.

Table S1. Slow heart rate in *smo* mutants

Genotype	Heart rate (bpm)	n
wild-type	116 ± 1	42
smo mutant	79 ± 2	41

smo mutants exhibited a significantly reduced average heart rate (beats per minute (bpm) ± standard error) compared to their wild-type siblings (p<0.0001) at 48 hpf. Additionally, a subset of *smo* mutants (3/41) exhibited arrhythmia.

Table S2. Slow heart rate in *laf* mutants

Genotype	Heart rate (bpm)	n
wild-type	100 ± 2	27
laf mutant	62 ± 4	27

laf mutants exhibited a significantly reduced average heart rate (± standard error) compared to their wild-type siblings (p<0.0001) at 36 hpf.

Table S3. Slow heart rate in *chd* mutants

Genotype	Heart rate (bpm)	n
wild-type	141 ± 2	16
chd mutant	118 ± 7	14

chd mutants exhibited a significantly reduced average heart rate (± standard error) compared to their wild-type siblings (p=0.0012) at 48 hpf.

Table S4. Antibodies used for immunofluorescence

Name	Source	Catalog #	Dilution
**Primary antibodies**			
MF20 hybridoma supernatant	DSHB	MF20	1:10
S46 hybridoma supernatant	DSHB	S46	1:10
Rabbit anti-Isl1 polyclonal	GeneTex	128201	1:2000
Rat anti-mCherry mAb, Alexa Fluor 594	ThermoFisher Scientific	M11240	1:400
**Secondary antibodies**			
Goat anti-Mouse IgG2b, TRITC	Southern Biotech	1090-03	1:100
Goat anti-Mouse IgG1, FITC	Southern Biotech	1070-02	1:100
Goat anti-Rabbit IgG (H+L), Alexa 488	ThermoFisher Scientific	A11008	1:500
Goat anti-Rabbit IgG (H+L), Alexa 647	ThermoFisher Scientific	A21245	1:500
Goat anti-Mouse IgG (H+L), Alexa 647	ThermoFisher Scientific	A21235	1:100
